# Strategies for Alleviating the Burden Experienced by Informal Caregivers of Persons With Severe Mental Disorders in Transitional Countries: Protocol for a Scoping Review

**DOI:** 10.2196/44268

**Published:** 2023-07-24

**Authors:** Olindah Silaule, Fasloen Adams, Nokuthula Gloria Nkosi

**Affiliations:** 1 Department of Occupational Therapy Faculty of Health Sciences University of the Witwatersrand Parktown South Africa; 2 Department of Occupational Therapy Stellenbosch University Tygerberg South Africa; 3 Department of Nursing Education University of the Witwatersrand Parktown South Africa

**Keywords:** burden, caregiver burden, caregiver care, caregiver intervention, caregiver stress, caregiver, developing country, guidelines, implementation strategy, informal carers, mental disorder, mental well-being, review method, rural, scoping review, support strategies

## Abstract

**Background:**

Caregiver burden is highly prevalent among the informal caregivers of persons with severe mental disorders (SMDs). As such, strategies to support informal caregivers are necessary to enable them to cope with their caregiving role. Currently, there is limited evidence on the extent of existing strategies for supporting informal caregivers of persons with SMDs in transitional countries.

**Objective:**

This study presents a scoping review protocol to identify and describe the extent and type of evidence on the existing strategies for alleviating caregiver burden among informal caregivers of persons with SMDs in transitional countries.

**Methods:**

This scoping review will be conducted using the Joanna Briggs Institute’s methodology for scoping reviews. The participants, concept, and context framework will be used to select relevant studies. This review will include studies on strategies for addressing caregiver burden among informal caregivers, with a specific focus on studies outlining caregiver interventions, caregiver support, and policies with strategies for supporting informal caregivers of persons with SMDs. Relevant studies conducted in transitional countries will be considered for inclusion. There will be no restrictions on publication type or design. Published literature will be accessed by searching electronic databases, including PubMed, MEDLINE, CINAHL, and PsycINFO; ProQuest will be used to access gray literature. Additionally, the reference lists of key studies will be reviewed to identify studies for inclusion. The search will be restricted to articles published between 2011 and 2021. Two reviewers will work independently to screen all abstracts and full texts for inclusion in line with the set inclusion criteria. Extracted data will be categorized and described using descriptive qualitative content analysis.

**Results:**

This protocol will guide a scoping review to identify and describe the extent and type of evidence on the existing strategies for alleviating caregiver burden among informal caregivers of persons with SMDs in transitional countries. The main results of this scoping review will synthesize evidence from peer-reviewed and gray literature sources outlining various services and interventions for supporting informal caregivers of people with SMDs in transitional countries. In addition, existing gaps in the literature will be identified to inform future studies.

**Conclusions:**

The increase in caregiver burden among informal caregivers in mental health warrants the development and implementation of strategies for alleviating the burden. This scoping review aims to increase awareness on the various services and intervention strategies for alleviating burden among informal caregivers in transitional countries.

**International Registered Report Identifier (IRRID):**

RR1-10.2196/44268

## Introduction

Severe mental disorders (SMDs) are disabling, chronic psychiatric conditions with a substantial burden on the patients and their caregivers [1]. These disorders include schizophrenia spectrum and other psychotic disorders, bipolar and related disorders, and depressive disorders [2]. People who are diagnosed with SMDs often exhibit long-term symptoms that compromise their ability to fulfill occupational and social roles [3]. For example, depressive disorder is characterized by prolonged feelings of sadness and guilt and reduced energy levels, which hinder participation in activities of daily living and work-related activities [4-7]. Those diagnosed with schizophrenia spectrum and other psychotic disorders often exhibit psychotic behavior, apathy, social withdrawal, and cognitive impairments, which all lead to a deterioration of social and occupational functioning [2,8]. Similarly, people with bipolar and related disorders are likely to experience considerable impairments at work and in their family lives and often struggle to form and maintain social relationships [9,10]. Given the chronic nature of SMDs, the symptoms are often persistent and thus require sustained care and management [11,12].

In resource-constrained settings in transitional countries, the care and management of those with SMDs relies on informal caregivers, making them instrumental in bridging the treatment gap [13]. Informal caregivers can be a family member, a friend, a neighbor, or any good Samaritan within the community responsible for providing care to a person with a chronic condition [14,15]. Informal caregivers assume responsibility for the health and well-being of those with mental disorders [16]. They are expected to manage the patient’s behavior, structure their routines, and monitor their treatment [17-19]. To fulfill these responsibilities, most informal caregivers are obligated to live with the mental health care user 24 hours a day, every day of the year, to support them [16]. Fulfilling the responsibilities of caregiving and dealing with the recurring symptoms of SMDs result in high caregiver burden, stress, a decline in health and well-being, and a decreased sense of support [18].

Informal caregivers of persons with SMDs require ongoing access to information, guidance, and support to enable them to cope with their caregiving responsibilities. Various strategies for supporting and alleviating caregiver burden among the informal caregivers of persons with SMDs have been proposed in developed countries, including the United States and the United Kingdom. These strategies include the provision of family-based interventions and support programs. Family-based interventions encompass different combinations of psychotherapeutic strategies, including psychoeducation, stress management, cognitive appraisal, and problem-solving skills [20].

Psychoeducation is described as a systematic and structured psychotherapeutic intervention that contains a brief introduction to the mental disorder, presenting symptoms, and information on signs of early relapse [21]. This intervention aims to inform persons with SMDs and their caregivers about the available treatment with details on efficacy, safety, common side effects, cost, and methods to identify burden and coping strategies [17]. To ensure effectiveness, psychoeducational sessions can initially be offered weekly and later once a month [15]. Similarly, cognitive behavioral therapy (CBT) involves training informal caregivers to develop cognitive and behavioral skills to enable them to cope with current stressors [20]. This makes CBT suitable for addressing the complex demands and stresses attached to the caregiving role [20]. The cognitive skills gained through CBT address dysfunctional thoughts that may arise from a negative appraisal of the caregiving role, and the behavioral skills aim to increase pleasure when performing the caregiving activities [21]. This intervention seeks to promote positive attitudes such as empathy, affective support, and changing verbal communication patterns between the caregivers and the care recipients [21]. CBT can be offered individually or in a group through diverse platforms, including face-to-face, web-based, and phone-based modes [22]. CBT has been found to be beneficial in reducing burden among caregivers of persons with schizophrenia [23].

Most family-based interventions have been developed and tested in Western countries. The American Psychiatric Association and the United Kingdom’s National Institute for Health and Care Excellence (NICE) guidelines for schizophrenia recognize the importance of family-based interventions for caregivers [24,25]. To ensure the effectiveness of these interventions, it is recommended that caregivers be involved in a minimum of 10 sessions over a period of 3 months to 1 year [26,27]. These interventions can be offered to caregivers individually or in a group session. Traditionally, a trained therapist or health care professional is required to offer these interventions to single or multiple families. Several randomized controlled trials conducted in the United States and the United Kingdom yielded positive results related to the effect of psychoeducation on families’ social function and caregiver burden in those with psychotic disorders [28]. Additionally, these interventions have been shown to increase the quality of life for caregivers and care recipients and are associated with positive patient outcomes, including increased medication compliance, which reduces relapses and hospitalization [14,29].

Peer-led support groups have been identified as another strategy to alleviate caregiver burden among the informal caregivers of persons with SMDs. Peer-led support groups, such as the National Alliance on Mental Illness (NAMI), were established in the United States as part of the larger social movement of self-help [30]. These support groups are meant to address the needs of service users and their caregivers that are not adequately addressed by standard health care services. They provide a platform through which informal caregivers can share and re-examine their own caregiving difficulties and challenges with other caregivers in similar situations. Additionally, it gives informal caregivers an opportunity to learn from others by exchanging information on SMDs and the experiences of caregiving [31]. Peer-led support groups are useful for improving caregiver attitudes, emotions, and empathy. Subsequently, they help improve effective communication and relationships among caregivers and their care recipients [32,33]. They are commended for being flexible and taking a more client-centered approach to enable caregivers to cope with the demands of their caregiving [28]. Previous studies conducted in the United States, the United Kingdom, and China demonstrated that peer-led support groups for informal caregivers of persons with SMDs can reduce caregiver burden and improve the caregivers’ knowledge and stress management [32,33].

Although most research on strategies for alleviating caregiver burden among informal caregivers originated in Western countries, including the United States and the United Kingdom, there has been a phenomenal increase in studies conducted in transitional countries [34]. There has been an emergence of randomized controlled trials in transitional countries, including China, India, Iran, Pakistan, Thailand, and Malaysia, over the last decade [31,35-38]. The growing body of knowledge from studies outlining strategies for alleviating caregiver burden among informal caregivers in transitional countries calls for a review of the literature in this area. Currently, no scoping reviews have been published on strategies for alleviating caregiver burden among informal caregivers of persons with SMDs. This scoping review will therefore present available evidence on strategies for alleviating informal caregiver burden. Published and gray literature from caregiver burden studies, caregiver intervention and caregiver support studies, and mental health policy documents will be reviewed to allow for the synthesis of available evidence in transitional countries.

## Methods

### Overview

This protocol is a systematic scoping review of the literature reporting on strategies for alleviating caregiver burden among the informal caregivers of persons with SMDs in transitional countries. A scoping review method was selected to present an outline of the evidence that exists in the area of interest and the gaps for further research [39]. The proposed scoping review will be conducted in accordance with the Joanna Briggs Institute’s (JBI) methodology for scoping reviews [39]. This methodology is based on 5 stages that guide the development of the protocol and the scoping review, which include identifying the research question and the relevant studies, selecting the studies, charting the data, and assembling the results to identify the relevant implications for policy, practice, and research [39].

### First Stage: Identifying the Research Question

The main research question is: “What strategies exist for alleviating caregiver burden among informal caregivers of persons with SMDs in transitional countries?”

### The Research Aims

The research aim is to present existing literature on strategies for alleviating caregiver burden among informal caregivers of persons with SMDs in transitional countries.

### The Research Objectives

This study has the following research objectives:

1. To identify existing strategies for alleviating caregiver burden among informal caregivers of persons with SMDs in transitional countries.
2. To describe the characteristics of the strategies used to alleviate caregiver burden among informal caregivers of persons with SMDs in transitional countries.

This study will use the participants, concept, and context (PCC) framework (Textbox 1) to guide the selection of relevant studies and ensure alignment with the research question.

**The participants, concept, and context (PCC) framework for eligibility of studies.**

Criteria and determinants

- Population
  - Informal caregivers of persons with severe mental disorders in transitional countries
  - Informal caregivers include family, friends, neighbors, or community members, often unpaid, to providing care
- Concept
  - Caregiver burden
  - Caregiver strain
  - Caregiver stress
  - Caretaker role fatigue
- Context
  - Transitional countries (including Africa, Asia, Latin America, and the Caribbean)
  - Lower- to middle-income countries

### Second Stage: Identifying the Relevant Studies

This scoping review will consider both published and unpublished studies detailing strategies for supporting informal caregivers in mental health in transitional countries. The 3-step search strategy process will be used to identify relevant studies. An initial limited search of PubMed, MEDLINE, CINAHL, and PsycINFO will be undertaken to identify relevant articles published over the last 10 years. This will be followed by the analysis of text words contained in the titles and abstracts of relevant articles and of the index terms used to describe the articles. The initial key words will include [informal caregiver/s OR caregiver/s] AND [caregiver burden OR caregiver stress] AND [support strategy/ies OR intervention/s] AND [severe mental disorder/s OR mental illness] AND [developing country/ies OR underdeveloped country/ies]. The second search will include all keywords and index terms identified and revised with the assistance of a librarian at the University of the Witwatersrand. The search strategy will be adapted for each included database and information source (Multimedia Appendix 1). For unpublished or gray literature, a targeted search of dissertations, theses, and conference abstracts will be conducted on “ProQuest Dissertations and Theses Global” and public health and conference databases. Lastly, the reference lists of all included sources of evidence will be screened for additional studies.

### Third Stage: Selection of Studies

The PCC framework (Textbox 1) provides detailed eligibility criteria to ensure that the content of relevant studies aligns with the research question (Textbox 2).

**Inclusion and exclusion criteria.**

**Inclusion criteria**

- Contain details on strategies for alleviating caregiver burden among the informal caregivers of persons with mental disorders in transitional countries as classified by the World Bank
- Contain strategies for informal caregivers, which includes family, friends, neighbors, or community members, often unpaid, to provide ongoing care to a person with a mental disorder
- Contain strategies for caregivers of persons with severe mental disorders (SMDs), including schizophrenia spectrum and other psychotic disorders, bipolar and related disorders, depressive disorders, and personality disorders
- Contain strategies targeted towards alleviating caregiver burden, caregiver strain, caregiver stress, and caregiver role fatigue
- Include documents on caregiver intervention and caregiver support and mental health policy documents outlining relevant strategies
- Were published in the last 10 years, that is, between 2011 and 2021
- Be primary and secondary studies with both quantitative, qualitative, and mixed methods research approaches

**Exclusion criteria**

- Those outlining strategies for supporting caregivers who are paid to provide care
- Strategies that focus only on care recipients
- Strategies for informal caregivers of persons with intellectual disabilities, Alzheimer disease, and dementia

The search strategy will be piloted to check the appropriateness of the identified keywords and databases. All eligible articles will be uploaded on Mendeley Reference Manager. The identified articles will then be uploaded to Covidence, and duplicates will be removed. Covidence is a web-based primary screening and data extraction tool used to streamline the systematic review process. The author will be responsible for searching and uploading the identified studies, and the author and co-reviewer will be responsible for screening the titles, abstracts, and full texts of the identified studies.

**Figure 1:**
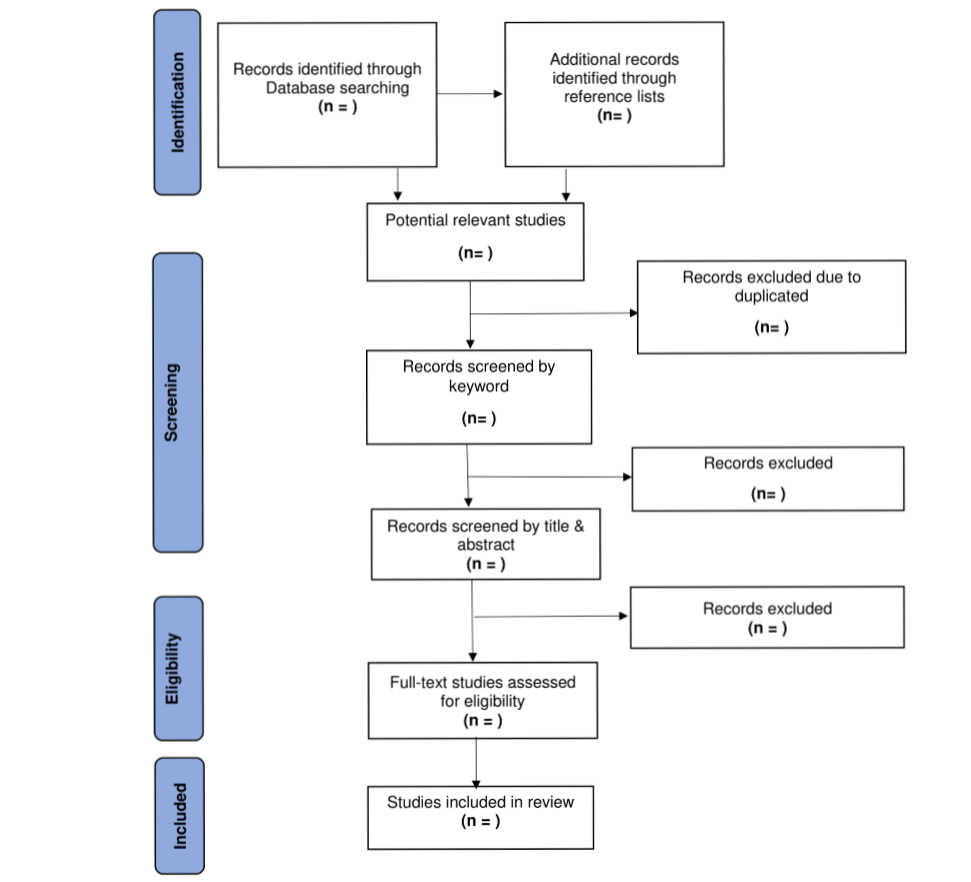
PRISMA (Preferred Reporting Items for Systematic Reviews and Meta-Analyses) flow diagram for the scoping review process [39].

The initial search and screening of titles and abstracts will commence in September 2021, and this will be followed by the second search and screening of titles, abstracts, and full texts in January-March 2022. In April-May 2022, the reviewers plan to conduct a full text review for additional studies identified in the references of the included studies. Throughout the process, all attempts will be made to obtain the full texts of the eligible studies by searching the University’s library guides and the internet and by collaborating with a librarian at the University of the Witwatersrand Faculty of Health Sciences. The author and identified co-reviewer will be responsible for conducting a full text review of the identified studies. All excluded studies, along with the reasons for exclusions, will be tracked on Covidence. All identified conflicts will be resolved through constant discussions between the 2 reviewers and a web-based meeting will be held biweekly. The degree of agreement between the reviewers will be calculated on Covidence and reported in the findings. The results of the search and the study inclusion process will follow the recommendations in the PRISMA-ScR (Preferred Reporting Items for Systematic Reviews and Meta-Analyses Extension for Scoping Reviews) checklist (Multimedia Appendix 2) [39]. This will be mapped using the PRISMA-P (PRISMA Extension for Protocols) chart (Figure 1).

### Fourth Stage: Charting the Data

The data will be charted using an electronic data extraction form [39] on the Covidence software. A total of 2 separate data extraction forms will be used for the research articles (Table S1 in Multimedia Appendix 3) and the policy and guideline documents (Table S2 in Multimedia Appendix 3). This is useful for capturing the relevant information from each included study.

A total of 2 independent reviewers based at separate tertiary institutions will carry out the data extraction. Communications regarding consensus will be facilitated through regular web-based meetings and face-to-face meetings when necessary. Before using the data extraction forms, piloting will be done to ensure the standardization and relevance of the extracted information. This process will be conducted at the end of the initial limited search phase of the scoping review. This process is essential to ensure efficient, meaningful extraction of data from all included papers. This process will be conducted at the end of the initial limited search phase of the scoping review. A sample of 10 included papers meeting the eligibility criteria will be selected to test the quality and validity of the data extraction tools [40]. First, the included studies will be purposely sampled, and second, data will be extracted from the included studies. The sample synthesis will then be conducted after the data analysis process, which is outlined in the data analysis and presentation. The results from the synthesis will be used to inform the modification of the data extraction form. This process is essential to ensure efficient and meaningful extraction of data from all included papers. To ensure the quality and validity of the modified data extraction form, the piloting process will be repeated at the end of the second search.

### Fifth Stage: Data Analysis and Presentation

A descriptive qualitative content analysis will be conducted inductively using NVIVO software to code the characteristics of the extracted data into overall categories [39]. This method of analysis is selected as it allows for rich meanings and insights to be derived from text by interpreting the manifest and latent content of the text, which is done through rigorous analysis and an understanding of a phenomenon’s critical processes [41]. The outcome of this scoping review will inform the development of interview guides for the subsequent studies, and therefore, a rigorous data analysis is required. A PRISMA-ScR flowchart (Figure 1) will be used to show the number of identified included and excluded studies. The various strategies for alleviating informal caregiver burden and their outcomes will be tabled under the different identified categories, including, but not limited to, the type of support strategies and interventions, the outcomes of the strategies and interventions used, and the challenges related to the implementation of these strategies and interventions [39]. Graphs and tables will be provided to enhance clarity. Existing gaps identified in the support strategies and interventions for informal caregivers in mental health, as well as the outcomes and challenges encountered will be identified to draw conclusions on the scoping review.

### Ethics Approval

This scoping review forms part of a PhD project aimed at developing guidelines to alleviate caregiver burden among informal caregivers of persons with SMDs in South Africa, a transitional country. Ethical clearance (M200957) was obtained from the University of the Witwatersrand Human Research Ethics Committee.

## Results

This protocol guides a scoping review aimed at identifying and describing the extent and type of evidence on the existing strategies for alleviating caregiver burden among informal caregivers of persons with SMDs in transitional countries. The initial search and screening of titles and abstracts commenced in September 2021, and this was followed by the second search and screening of titles, abstracts, and full texts from January to March 2022. A full-text review of the additional studies identified from the included studies was conducted from April to June 2022. Overall, 44 studies were identified for inclusion in the main study. The data were analyzed and written up for publication in September 2022. The scoping review article was submitted for consideration in a peer-reviewed journal, and the authors expect publication by the end of 2023. The results of this scoping review are useful to policy makers, mental health managers, health care professionals, and informal caregivers in mental health since they present the services and practical strategies that can be implemented to alleviate caregiver burden among informal caregivers. Additionally, existing gaps in the literature have been identified to inform future studies.

## Discussion

### Principal Findings

This protocol will guide a scoping review to identify and describe the extent and type of evidence on the existing strategies for alleviating caregiver burden among informal caregivers of persons with SMDs in transitional countries. There is acknowledgment of the unique role informal caregivers play in the care and management of people with SMDs within transitional countries and the subsequent burden arising from occupying this role [13,42,43]. A supported informal caregiver is likely to provide quality care, which is critical for the recovery of a person with a SMD [44]. The synthesis and analysis of existing literature on the various services and intervention strategies for alleviating caregiver burden forms a basis through which the development and implementation of these strategies can be facilitated within transitional countries where such evidence is lacking. Three researchers who have knowledge and experience in the JBI scoping review methodology developed the protocol. A librarian helped develop a comprehensive search strategy to allow for broad access to existing literature sources. To the best of our knowledge, there is no synthesis of literature in this area; this review therefore adds to the body of knowledge on the support for informal caregivers in mental health.

### Limitations

The scoping review is limited to studies conducted in transitional countries; thus, the scope excludes studies conducted in developed countries, limiting the extent of the evidence on the strategies for supporting informal caregivers in mental health. Additionally, the review only includes studies conducted in English, creating a bias in the scope of the collated evidence. While the scoping review intends to include both peer reviewed and gray literature, it may not cover the entire existing evidence; as a result, studies in indexed databases may not be identified. The current review focuses on strategies for alleviating caregiver burden in SMDs, which locates it within the traditional Western medical health system paradigm. It is critical to acknowledge culture-bound syndromes, particularly within transitional countries; however, in other transitional countries, these syndromes are still classified under SMDs, which is within the Western medical terminology.

### Conclusions

Informal caregivers are the backbone of recovery for persons with SMDs, and thus, strategies for alleviating their caregiver burden are critical, especially in resource-constrained transitional countries. With the growing research on the need for strategies aimed at supporting informal caregivers in their role, it is necessary to understand the extent of the burden and characteristics of these strategies in transitional countries. This scoping review will generate evidence to raise awareness on the various services and intervention strategies that can be developed and implemented to alleviate burden among informal caregivers in mental health within transitional countries.

## Appendix

Multimedia Appendix 1 Search terms.

Multimedia Appendix 2 Preferred Reporting Items for Systematic Reviews and Meta-analyses extension for scoping review (PRISMA-ScR) checklist.

Multimedia Appendix 3 Data extraction tables adapted from Peters et al [39].

## Abbreviations

CBT: cognitive behavioral therapy
JBI: Joanna Briggs Institute
NAMI: National Alliance on Mental Illness
NICE: National Institute for Health and Care Excellence
PCC: participants, concept, and context
PRISMA-P: Preferred Reporting Items for Systematic Reviews and Meta-Analyses Extension for Protocols
PRISMA-ScR: Preferred Reporting Items for Systematic Reviews and Meta-Analyses Extension for Scoping Reviews
SMD: severe mental disorder

## Data Availability

The data sets generated during and analyzed during this study are available from the corresponding author on reasonable request.
