# Supplementary figures and images for: Strategies for Alleviating the Burden Experienced by Informal Caregivers of Persons With Severe Mental Disorders in Transitional Countries: Protocol for a Scoping Review
Source: JMIR Res Protoc. 2023 Jul 24;12:e44268. doi: 10.2196/44268 (PMC10407773; doi:10.2196/44268)

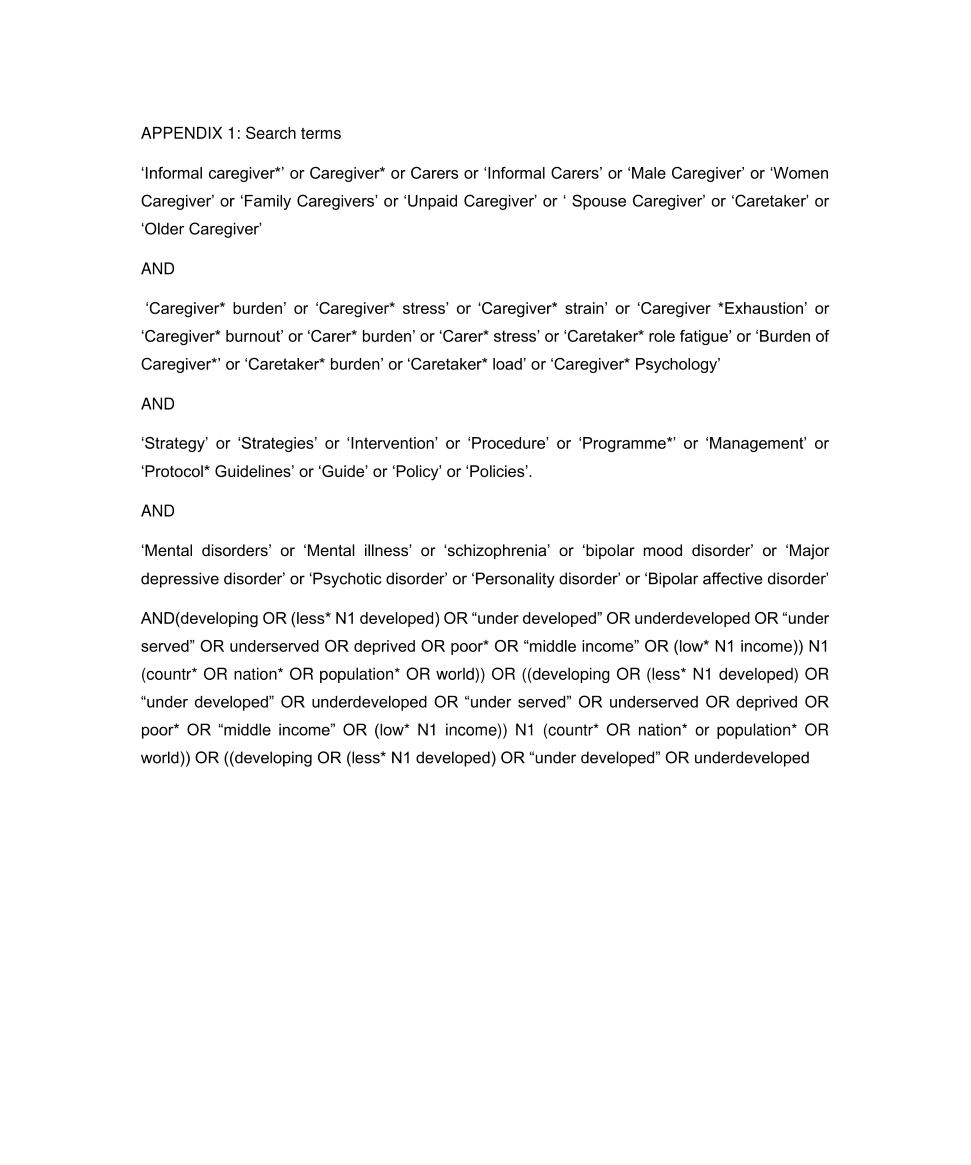

Supplement: Multimedia Appendix 1 [file resprot_v12i1e44268_app1.png]
