# Supplementary material for: Strategies for Alleviating the Burden Experienced by Informal Caregivers of Persons With Severe Mental Disorders in Transitional Countries: Protocol for a Scoping Review
Source: JMIR Res Protoc. 2023 Jul 24;12:e44268. doi: 10.2196/44268 (PMC10407773; doi:10.2196/44268)
Supplement: Multimedia Appendix 3 [file resprot_v12i1e44268_app3.docx]

**Table S1**. Data extraction table adapted from Peters et al [39].

| Note: *When completing your data extraction sheet be aware that all information that is not reported should be indicated as such. No area should be left empty. Indicate, “not reported” if the information is not reported on. Do not assume information, and always report as it is, e.g., with gender if it says 80% was male and do not report on the other 20%, do not assume it was female.* | | | |
| --- | --- | --- | --- |
| Scoping Review Details: | | | |
| Scoping Review Title | | |  |
| Scoping Review Objective/s | | |  |
| Scoping Review question/s | | |  |
| General information: | | | |
| Study ID | | |  |
| Study title | | |  |
| Lead Author (surname and initials of the lead author) | | |  |
| **Inclusion/Exclusion Criteria:** | | | |
| Population | | |  |
| Concept | | |  |
| Context (country) | Developing country (incl. Africa, Asia and Latin America and the Caribbean) | |  |
|  | Underdeveloped country | |  |
|  | Mixed countries | |  |
| Context  (type of setting) | Rural | |  |
|  | Urban | |  |
|  | Peri-urban | |  |
|  | Not specified | |  |
| Type/s of evidence source: | | | |
| Journal article | | |  |
| Research report | | |  |
| Guideline document | | |  |
| Opinion piece | | |  |
| Research thesis | | |  |
| **Evidence Source Details and Characteristics:** | | | |
| **Aim/s of the study** | | |  |
| Citation details | Author/s | |  |
|  | Date of publication | |  |
|  | Journal | |  |
|  | Volume/Issue/Article Number | |  |
|  | Pages | |  |
| Country (exact country where the study took place) | | |  |
| Context (specify the setting where the study took place) | | |  |
| Research approach | Quantitative | |  |
|  | Qualitative | |  |
|  | Mixed methods | |  |
|  | Multimethod | |  |
| Study design | Randomised controlled trial | |  |
|  | Non-randomised experimental study | |  |
|  | Cohort study | |  |
|  | Cross-sectional study | |  |
|  | Case control study | |  |
|  | Qualitative research | |  |
|  | Quantitative research | |  |
|  | Prevalence study | |  |
|  | Case series | |  |
|  | Case report | |  |
|  | Clinical prediction rule | |  |
|  | Practice guideline | |  |
|  | Text and opinion | |  |
|  | Other | |  |
| Study funding sources | | |  |
| Possible conflicts of interest for study authors | | |  |
| Participants details: | | | |
| Age of caregiver (comment if mixed age) | | |  |
| Age of care recipient (comment if mixed age) | | |  |
| Gender of caregiver | Female | |  |
|  | Male | |  |
|  | Mixed gender groups (male and female) | |  |
| Gender of care recipient | Female | |  |
|  | Male | |  |
|  | Mixed gender groups (male and female) | |  |
| Diagnosis of the care recipient | | |  |
| Sampling size | | |  |
| Total number of participants | | |  |
| Intervention type: | | | |
| Intervention content | Type of intervention | |  |
|  | Who developed the intervention | |  |
|  | Who delivered the intervention | |  |
|  | Type of burden targeted by the intervention (objective, subjective etc.) | |  |
| Intervention description | | Duration of intervention |  |
|  |  | Number of sessions (overall) |  |
|  |  | Number of sessions (over weeks) |  |
|  |  | Location for intervention |  |
|  |  | Measurement instruments |  |
| **Conclusion and recommendations extracted from source of evidence:** | | | |
| Conclusions | | |  |
| Recommendations | | |  |

**Table S2**. Data extraction table for policy documents adapted from Peters et al [39].

| Scoping Review Details: | | | | |
| --- | --- | --- | --- | --- |
| Scoping Review Title | | | |  |
| Scoping Review Objective/s | | | |  |
| Scoping Review question/s | | | |  |
| General information: | | | | |
| Study ID | | | |  |
| Study title | | | |  |
| Lead Author (*surname and initials of the lead author)* | | | |  |
| **Inclusion/Exclusion Criteria:** | | | | |
| Population | | | |  |
| Concept | | | |  |
| Context (country) | | Developing country (incl. Africa, Asia and Latin America and the Caribbean) | |  |
|  |  | Underdeveloped country | |  |
|  |  | Mixed countries | |  |
| Context  (type of setting) | | Rural | |  |
|  |  | Urban | |  |
|  |  | Peri-urban | |  |
|  |  | Not specified | |  |
| Type/s of evidence source: | | | | |
| Public health policy | | | |  |
| Mental health policy | | | |  |
| Guideline document | | | |  |
| **Evidence Source Details and Characteristics:** | | | | |
| **Aim/s of the policy document** | | | |  |
| **Objective/s of the policy document** | | | |  |
| Citation details | Author/s | | |  |
|  | Date of publication | | |  |
|  | Pages | | |  |
| Country | | | |  |
| Context (urban/peri-urban/rural) | | | |  |
| Target population: | | | | |
| Age of caregiver (comment if mixed age) | | | |  |
| Age of care recipient (comment if mixed age) | | | |  |
| Gender of caregiver | | Female | |  |
|  |  | Male | |  |
|  |  | Mixed gender groups (male and female) | |  |
| Gender of care recipient | | Female | |  |
|  |  | Male | |  |
|  |  | Mixed gender groups (male and female) | |  |
| Population (*description of the population that the policy is targeted towards)* | | | |  |
| Characteristics of policy document: | | | | |
| Execution plan | | | |  |
| Defined procedure | | | |  |
| Periodical review | | | |  |
| Intervention type: | | | | |
| Intervention content | | Type of intervention | |  |
|  |  | Who developed the intervention | |  |
|  |  | Who delivered the intervention | |  |
|  |  | Type of burden targeted by the intervention (objective, subjective etc) | |  |
| Intervention description | | | Duration of intervention |  |
|  |  |  | Number of sessions (overall) |  |
|  |  |  | Number of sessions (over weeks) |  |
|  |  |  | Location for intervention |  |
|  |  |  | Measurement instruments |  |
| Targeted output | | | |  |
| **Recommendations extracted from source of evidence:** | | | | |
| Conclusions | | | |  |
| Recommendations | | | |  |
